# Metabolic syndrome is associated with accelerated brain aging

**DOI:** 10.1002/alz.71563

**Published:** 2026-07-14

**Authors:** Abigail Dove, Jiao Wang, Rongrong Yang, Sakura Sakakibara, Zoe Arvanitakis, Andrea L. C. Schneider, Rebecca F. Gottesman, Weili Xu

**Affiliations:** ^1^ Aging Research Center Department of Neurobiology Care Sciences and Society Karolinska Institutet Stockholm Sweden; ^2^ Division of Clinical Geriatrics Department of Neurobiology Care Sciences and Society Karolinska Institutet Stockholm Sweden; ^3^ Department of Neurology University of Pennsylvania Perelman School of Medicine Philadelphia Pennsylvania USA; ^4^ National Clinical Research Center for Geriatrics West China Hospital Sichuan University Chengdu China; ^5^ Public Health Science and Engineering College Tianjin University of Traditional Chinese Medicine Tianjin China; ^6^ Rush Alzheimer's Disease Center Rush University Medical Center Chicago Illinois USA; ^7^ Department of Biostatistics Epidemiology, and Informatics University of Pennsylvania Perelman School of Medicine Philadelphia Pennsylvania USA; ^8^ National Institute of Neurological Disorders and Stroke Intramural Research Program National Institutes of Health Bethesda Maryland USA

**Keywords:** brain age gap, brain aging, biomarkers, metabolomics, metabolic syndrome

## Abstract

**INTRODUCTION:**

Metabolic syndrome (MetS) is associated with increased dementia risk, but its relationship with brain aging is unclear.

**METHODS:**

The study included 27,375 UK Biobank participants aged 40 to 70 years. MetS was defined as having at least three of five components: central adiposity, hypertension, dyslipidemia, hypertriglyceridemia, and hyperglycemia. Levels of 33 plasma metabolites were measured from baseline blood samples. Brain age was estimated using a machine learning model based on 1079 phenotypes from brain magnetic resonance imaging (MRI) scans and used to calculate brain age gap (BAG, i.e., brain age minus chronological age).

**RESULTS:**

Participants with MetS had significantly higher BAG compared to MetS‐free individuals (*β* = 1.13; 95% confidence interval [CI]: 0.99 to 1.27). Each individual MetS component was also associated with higher BAG. Eight metabolites significantly mediated the MetS–BAG association (mediation proportion: 2.6% to 16.5%), including apolipoproteins, fatty acids, and inflammatory markers.

**DISCUSSION:**

MetS is associated with accelerated brain aging, partly mediated by inflammation and altered lipid metabolism.

## Highlights

- We examined the association between MetS and brain aging.
- Having more MetS components was dose‐dependently related to older brain age.
- Inflammatory, lipid, and fatty acid metabolites partially mediated this association.
- Findings suggest that MetS may represent a modifiable target to slow brain aging.

## 1 BACKGROUND

Metabolic syndrome (MetS) is a cluster of interrelated cardiometabolic risk factors that come together in a single individual, including obesity, hypertension, hyperglycemia, high triglycerides, and low HDL cholesterol.^1^ An estimated 25% of the global adult population has MetS, defined as the coexistence of ≥3 of these components, and prevalence rises sharply with age.^1^ Emerging evidence has emphasized the connection between MetS and brain health, linking MetS to cognitive impairment^2^ and decline^3^ as well as increased risk of neurological disorders such as stroke,^4^, ^5^ dementia,^6^, ^7^, ^8^ and Parkinson's disease.^9^, ^10^, ^11^, ^12^ One way that MetS impacts brain health may be via changes in neuroimaging markers. Some magnetic resonance imaging (MRI) studies have linked MetS to lower brain volume and a higher burden of white matter hyperintensities,^13^, ^14^, ^15^, ^16^ while others have reported no such associations.^17^, ^18^

In recent years, modeling methods have been introduced to estimate “brain age” based on subtle variations in several different structural brain MRI features such as regional brain volumes, cortical thickness, and white matter integrity.^19^ Brain age gap (BAG) refers to the discrepancy between an individual's brain age and their chronological age.^19^ Having an older‐appearing brain relative to one's chronological age – i.e., high BAG – can be a sign of departure from the normal aging process and has been associated with significantly elevated risk of cognitive decline, dementia, and other neurological disorders.^19^ A growing literature has linked individual MetS components such as diabetes,^20^, ^21^, ^22^, ^23^, ^24^, ^25^, ^26^, ^27^ elevated blood pressure,^23^, ^24^, ^28^ and obesity^23^, ^24^, ^29^ to older brain age. However, these components are rarely present in isolation and may interact in complex ways to affect brain health. The relationship between the coexistence of multiple cardiometabolic risk factors and brain age has yet to be addressed.

Similarly, the biological mechanisms linking MetS and brain health remain poorly understood. Nuclear magnetic resonance (NMR) metabolomics enables the quantification of a broad array of small molecules in plasma, making it possible to identify biochemical signatures associated with a phenotype of interest.^30^ Previous studies identified distinct signatures of circulating metabolites that characterize MetS, including amino acids, carbohydrates, fatty acids, peptides, and steroids.^31^ However, whether and to what extent these circulating metabolites mediate the relationship between MetS and brain‐related outcomes has not been explored to date.

This study investigated the relationship between MetS and brain aging and its underlying biological mechanisms, leveraging detailed neuroimaging and metabolite biomarker data from >34,000 middle‐aged and older adults from the UK Biobank. Specifically, we aimed to (1) estimate the association of MetS and its components with BAG and (2) identify plasma metabolites that mediate the MetS‐BAG association.

## 2 METHODS

### 2.1 Study design and population

The UK Biobank is an ongoing longitudinal study that includes over 500,000 adults between the ages of 40 and 70 from across the United Kingdom.^32^ The baseline examination took place between 2006 and 2010 at one of 22 assessment centers across the country and consisted of clinical assessments, cognitive tests, questionnaires on sociodemographic and lifestyle factors, and collection of blood samples. Approximately 9 years later, between 2014 and 2020, all surviving participants were invited to return for a brain MRI scan.

Selection of the study population is illustrated in Figure S1. The analysis was first restricted to 34,295 participants who underwent brain MRI scans and had complete information available on all imaging phenotypes. We then excluded 667 participants with neurological disorders (including, for example, dementia or stroke; Table S1) at the time of the MRI scan and 6253 with missing information on one or more MetS components, leaving a sample of 27,375 for the analysis of the association between MetS and BAG. Subsequent analyses to identify metabolite mediators of the MetS–BAG association were conducted in a subset of 16,904 participants with available data on plasma metabolites.

All data used in this study were obtained from the UK Biobank through application 67048 (PI: Weili Xu). Data collection procedures were approved by the UK National Research Ethics Service (Ref 11/NW/0382), and use of the data for the present analyses was additionally approved by the Regional Ethical Review Board in Stockholm (2024‐00520‐01). All subjects provided informed consent, and study procedures were conducted in accordance with the ethical standards put forth in the 1964 Declaration of Helsinki and its later amendments.

### 2.2 Assessment of MetS

MetS was defined using a modified version of the 2009 Harmonized Criteria from the International Diabetes Federation and American Heart Association.^33^ Participants were classified as having MetS if they had at least three of the following components directly measured during the baseline examination: (1) central adiposity (waist circumference ≥102 cm in males, ≥88 cm in females); (2) elevated blood pressure (≥130 mmHg systolic, ≥85 mmHg diastolic, or use of antihypertensive drugs); (3) hyperglycemia (HbA1c ≥5.7% or use of antidiabetic drugs); (4) elevated triglycerides (≥150 mg/dL); or (5) low HDL (<40 mg/dL in males, <50 mg/dL in females). In line with previous UK Biobank studies on MetS,^8^, ^14^, ^34^ we used HbA1c ≥5.7% instead of fasting glucose ≥100 mg/dL as the threshold for hyperglycemia, given that the UK Biobank study protocol did not require participants to fast before blood samples were collected.

In addition to the presence or absence of MetS, participants were also categorized according to their total number of MetS components (0 to 5), the presence or absence of each of the five components, and different possible constellations of the MetS components (2^5^ = 32 possible combinations in total).

**RESEARCH IN CONTEXT**

1. **Systematic review**: The literature was reviewed using traditional sources like PubMed. MetS has been consistently associated with increased risk of cognitive decline, dementia, and other neurological disorders. However, less is known about how MetS relates to brain aging. In addition, few studies have examined the pathways through which MetS may influence brain aging.
2. **Interpretation**: In a large neuroimaging cohort, MetS and all five of its constituent components were related to older brain age in relation to chronological age, and the gap between brain age and chronological age increased with the co‐occurrence of more MetS components. Plasma metabolites reflecting inflammation, apolipoproteins, and fatty acid metabolism partially mediated this association, suggesting possible biological pathways through which MetS may accelerate brain aging.
3. **Future directions**: Future studies should investigate whether improvements in metabolic health are associated with a deceleration in the pace of brain aging.

### 2.3 Brain MRI acquisition

Brain MRI scans were performed using Siemens Skyra 3T scanners a mean of 8.9 ± 1.8 (range: 4.3 to 12.9) years after the baseline assessment. The UK Biobank brain MRI acquisition and processing protocols have been detailed elsewhere^35^, ^36^ and are summarized in Table S2. A total of 1079 imaging‐derived phenotypes (IDPs) were extracted across a total of six brain MRI modalities: 165 from T1‐weighted MRI, one from T2‐fluid‐attenuated inversion recovery (FLAIR), 14 from T2* gradient echo, 675 from diffusion MRI, 210 from resting‐state functional MRI (fMRI), and 14 from task fMRI. Briefly, T1‐weighted imaging assesses regional brain volumes; T2‐FLAIR detects white matter hyperintensities indicative of vascular injury; T2* identifies microbleeds; diffusion MRI captures white matter microstructural integrity through fractional anisotropy and mean diffusivity; resting‐state fMRI evaluates intrinsic functional connectivity during rest; and task‐based fMRI does so during active engagement – in this case, a face/shape‐matching task.

### 2.4 Estimation of brain age and brain age gap

The procedure for brain age estimation was outlined in our previous studies^27^, ^37^, ^38^, ^39^, ^40^ and is elaborated in detail in Supporting Information: Methods 1. First, a subset of healthy participants was identified and randomly divided at a 4:1 ratio into a training set and validation set. Next, nine alternative machine learning models were trained and optimized to estimate brain age in the training set, based on Z‐scores for all IDPs. The candidate machine learning models were composed of three alternative regression models (least absolute shrinkage and selection operator [LASSO] regression, eXtreme Gradient Boosting [XGBoost], and support vector regression [SVR]) combined with three possible feature selection strategies (no feature selection, FeatureWiz, or recursive feature elimination with cross‐validation [RFECV]). All nine models were then applied to the validation set to compare their performance. The LASSO model without feature selection achieved the lowest mean absolute error (MAE) (Table S3) and was therefore chosen to predict brain age for the entire sample of participants with complete data available on all IDPs. In this model, 285 IDPs contributed significantly to the brain age estimate (Table S4). In the final step, a regression approach was used to correct the brain age estimates for age bias, given the tendency of brain age models to overpredict brain age in younger subjects and underpredict brain age in older subjects (Figure S2). Validation steps were taken to ensure age bias removal (Figure S3).

BAG represents the difference between an individual's estimated brain age and that individual's chronological age, calculated as follows: *BAG = brain age − age_time of MRI_*. Positive values for BAG indicate a brain that is older (i.e., less healthy), and negative values for BAG indicate a brain that is younger (i.e., healthier) than expected based on the individual's chronological age.

### 2.5 Quantification of plasma metabolites

A panel of 249 metabolites were quantified by NMR spectroscopy from plasma samples collected during the baseline examination using the Nightingale Health (Helsinki, Finland) NMR biomarker profiling platform.^41^ To enhance the translatability of our results, we focused on a set of 37 metabolites from the Nightingale Health platform that have been clinically validated for diagnostic use.^41^ After excluding four metabolites already encompassed in the definition of MetS (HbA1c, triglycerides, HDL, and total cholesterol), the present study included 33 clinically validated biomarkers. These are summarized in Table S5 and include cholesterols, apolipoproteins, fatty acids and fatty acid ratios, amino acids, indicators of fluid balance, glycolysis end products, and inflammatory markers.

### 2.6 Assessment of covariates

Information on the following covariates was collected during the baseline examination:

#### 2.6.1 Sociodemographic factors

Education level was self‐reported and dichotomized according to whether participants had completed college/university. Race and ethnicity were self‐reported according to the 2001 UK census categories and dichotomized as White versus non‐White (including Asian, Black, multiracial, or other). Socioeconomic status (SES) was assessed using the Townsend Deprivation Index (TDI), a measure of neighborhood‐level socioeconomic deprivation based on the prevalence of unemployment, household overcrowding, and car/home ownership in a given ZIP code of residence (higher TDI indicates greater socioeconomic deprivation).^42^

#### 2.6.2 Lifestyle behaviors

Smoking status was categorized as non‐smoking, former smoker, or current smoker according to self‐report. Participants self‐reported their weekly and monthly consumption of different types of alcoholic beverages (e.g., red wine, white wine, champagne, spirits, beer/cider, fortified wine), and this information was used to calculate average grams of alcohol consumed per week.^43^ Physical activity was measured using the International Physical Activity Questionnaire and classified as inactive (<600 metabolic equivalent [MET]‐min/week), moderate (600 to <3000 MET‐min/week), or active (≥3,000 MET‐min/week).^44^

#### 2.6.3 Alzheimer's disease‐related polygenic risk

Alzheimer's disease (AD)‐related polygenic risk score (PRS_AD_) was obtained from the UK Biobank's Standard PRS Set and represents the Z‐standardized sum of each participant's number of AD‐related alleles (including the well‐known *APOE* ɛ4 polymorphism) weighted by the strength of each allele's association with AD.^45^

### 2.7 Statistical analysis

Baseline characteristics of the study participants by MetS status were assessed using *t*‐tests for continuous variables and one‐way analysis of variance (ANOVA) for categorical variables.

Linear regression models were used to estimate β‐coefficients and 95% confidence intervals (CIs) for the associations between MetS, number of MetS components, different possible constellations of MetS components, and the presence of each individual MetS component with BAG. Least squares (LS) means of BAG (in years) were also estimated from the margins of the linear regression models. To assess departure from a simple linear trend, we additionally modeled the association between each MetS component and BAG using restricted cubic splines with 3 knots at fixed percentiles (10th, 50th, and 90th) of the distributions for waist circumference, systolic and diastolic blood pressure, HbA1c, triglycerides, and HDL. Compatibility of the data with a curvilinear relationship was assessed by testing the hypothesis that the coefficient of the second spline is zero, that is, when the cubic spline transformation of the predictor does not improve the fit of the data.

Next, separate linear regression models were used to identify which of the 33 metabolite biomarkers were significantly associated with MetS and which were significantly associated with BAG. Metabolites that were significantly associated with both MetS and BAG were then incorporated into generalized structural equation models (GSEMs) to analyze their mediating role in the association between MetS and BAG. Indirect effects (i.e., the influence of MetS on BAG that works through the metabolite of interest) were estimated with confidence intervals through bias‐corrected bootstrapping (500 replications). To account for multiple comparisons, a stricter *p* value of <0.0015 was used as the threshold for statistical significance in all analyses of plasma metabolite data (*p*_0.05_ / 33 metabolites = *p*_0.0015_). Patterns of variation between significant metabolites were subsequently visualized using principal component analysis (PCA), and Spearman correlations were used to explore between‐metabolite relationships.

Models were adjusted for age, sex, education level, race/ethnicity, SES, smoking, alcohol consumption, physical activity level, and PRS_AD_. Covariate adjustment strategy is illustrated in Figure S4. Missing values for covariates were imputed using multiple imputation by chained equations. The imputation model included all covariates previously described; estimates were pooled from 10 iterations.

In sensitivity analysis, we repeated the main analyses (1) using BAG calculated based on brain age estimates from the other candidate machine learning models, (2) using non‐imputed covariate data, and (3) after excluding participants with low cognitive performance (i.e., baseline cognitive test scores <25th percentile; Supporting Information: Methods 2) to minimize the possibility of reverse causality. We additionally evaluated the roles of sex and age in the MetS–BAG association through stratified analyses.

All analyses were performed using Stata SE 16.0 (Stata Corp, College Station, TX, USA).

## 3 RESULTS

### 3.1 Baseline characteristics of study sample

Baseline characteristics of the 27,375 study participants (mean age 54.9 ± 7.5; 52.3% female) are presented in Table 1. At baseline, 7800 (28.5%) participants met the criteria for MetS. The most prevalent MetS component was elevated blood pressure (66.8%), followed by low HDL cholesterol (36.5%), elevated triglycerides (35.9%), central adiposity (24.7%), and hyperglycemia (14.0%). Compared to those who were MetS‐free, participants with MetS tended to be older, male, of white race, and non‐college/university educated, were more likely to smoke and consume alcohol, and were less likely to be physically active. Participants with MetS were also more likely to have a higher PRS_AD_ and to carry at least one copy of the *APOE* ɛ4 allele. Similar results were observed when participants were instead grouped according to the number of MetS components present (Table S6).

**TABLE 1:** Baseline characteristics of the 27,375 study participants by MetS status.

		By MetS status	
Characteristics	Full sample (*n* = 27,375)	MetS‐free (*n =* 19,575)	MetS (*n =* 7800)
**Age, years**			
At baseline	54.9 ± 7.5	54.1± 7.5	57.0 ± 7.1*
At time of brain MRI	63.9 ± 7.6	63.1 ± 7.6	65.9 ± 7.3*
**Sex**			
Female	14,309 (52.3)	11,112 (56.8)	3197 (41.0)*
Male	13,066 (47.7)	8463 (43.2)	4603 (59.0)*
**College/university‐educated**	12,660 (46.4)	9508 (48.7)	3152 (40.6)*
**White race**	25,386 (93.0)	18,110 (92.8)	7276 (93.5)*
**Townsend Deprivation Index**	−1.92 ± 2.71	−1.96 ± 2.68	−1.81 ± 2.77*
**Smoking**			
Non‐smoker	16,619 (60.8)	12,370 (63.3)	4249 (54.6)*
Former smoker	9049 (33.1)	6069 (31.1)	2980 (38.3)*
Current smoker	1653 (6.1)	1102 (5.6)	551 (7.1)*
**Alcohol (g/week)**	139 ± 127	136 ± 121	147 ± 140*
**Physical activity**			
Inactive	4305 (18.2)	2766 (16.3)	1539 (23.0)*
Moderate	9921 (41.9)	7017 (41.4)	2904 (43.4)*
Active	9429 (39.9)	7179 (42.3)	2250 (33.6)*
***APOE* ε4 carrier**	6450 (27.7)	4580 (27.3)	1870 (28.7)*
**PRS_AD_**	0.04 ± 0.99	0.03 ± 0.98	0.07 ± 1.00*
**Brain age, years**	64.3 ± 9.1	63.2 ± 8.9	67.1 ± 9.1*
**BAG, years**	0.5 ± 5.1	0.1 ± 4.9	1.3 ± 5.6*
**Individual MetS components**			
Central adiposity	6770 (24.7)	2058 (10.5)	4712 (60.4)*
Elevated blood pressure	18,298 (66.8)	10,940 (55.9)	7358 (94.3)*
Hyperglycemia	3833 (14.0)	958 (4.9)	2875 (36.9)*
Elevated triglycerides	9834 (35.9)	3832 (19.6)	6002 (77.0)*
Low HDL cholesterol	9977 (36.5)	3707 (18.9)	6270 (80.4)*

*Note*: Data are presented as means ± standard deviations or number (proportion, %). Missing data: 81 (0.3%) for education level; 77 (0.3%) for race; 24 (<0.1%) for Townsend Deprivation Index; 54 (0.2%) for smoking status; 3328 (12.2%) for alcohol consumption; 3720 (13.6%) for physical activity level; 4065 (14.8%) for APOE ε4 status; 209 (0.8%) for PRS_AD_.

*Indicates significant (*p* value <0.05) pairwise comparison (reference group = MetS‐free)

### 3.2 MetS, MetS components, and BAG

Table 2 summarizes the associations of MetS and its components with BAG. The presence of MetS was associated with significantly higher BAG (*β* = 1.13 [0.99, 1.27]), such that brain age was on average 1.26 years older than chronological age among participants with MetS. We further observed a dose response relationship between higher number of MetS components present and higher BAG (*β* = 0.52 [0.47, 0.57] per each additional MetS component), with BAG rising as high as 2.30 years among participants with all five MetS components.

**TABLE 2:** Associations between MetS and brain age gap (BAG): results from linear regression models.

	n	BAG				LS mean (years)
		Basic‐adjusted		Multi‐adjusted		
		*β* (95% CI)	*p* value	β (95% CI)	*p* value	
**MetS status**						
MetS‐free	19,575	Reference		Reference		0.13 ± 0.04
MetS	7800	1.15 (1.02, 1.29)	<0.001	1.13 (0.99, 1.27)	<0.001	1.26 ± 0.06
**Number of MetS components**						
0	4,850	Reference		Reference		−0.48 ± 0.08
1	7,955	0.63 (0.45, 0.82)	<0.001	0.61 (0.43, 0.79)	<0.001	0.13 ± 0.06
2	6,770	1.06 (0.89, 1.26)	<0.001	1.03 (0.83, 1.22)	<0.001	0.54 ± 0.06
3	4,710	1.49 (1.28, 1.70)	<0.001	1.44 (1.23, 1.66)	<0.001	0.96 ± 0.07
4	2,363	2.20 (1.94, 2.46)	<0.001	2.16 (1.91, 2.42)	<0.001	1.68 ± 0.10
5	727	2.83 (2.43, 3.23)	<0.001	2.78 (2.38, 3.18)	<0.001	2.30 ± 0.19
*Continuous*		0.52 (0.47, 0.57)	<0.001	0.51 (0.46, 0.56)	<0.001	
**Individual MetS components**						
Central adiposity						
No	20,605	Reference		Reference		0.22 ± 0.04
Yes	6770	1.01 (0.87, 1.15)	<0.001	0.94 (0.80, 1.08)	<0.001	1.16 ± 0.06
Elevated blood pressure						
No	9077	Reference		Reference		−0.28 ± 0.06
Yes	18,298	1.17 (1.04, 1.31)	<0.001	1.10 (0.97, 1.24)	<0.001	0.82 ± 0.04
Hyperglycemia						
No	23,542	Reference		Reference		0.31 ± 0.03
Yes	3833	1.07 (0.89, 1.24)	<0.001	1.08 (0.90, 1.25)	<0.001	1.38 ± 0.08
Elevated triglycerides						
No	17,541	Reference		Reference		0.29 ± 0.04
Yes	9834	0.49 (0.36, 0.62)	<0.001	0.48 (0.34, 0.61)	<0.001	0.76 ± 0.05
Low HDL cholesterol						
No	17,398	Reference		Reference		0.20 ± 0.04
Yes	9977	0.68 (0.56, 0.81)	<0.001	0.72 (0.58, 0.85)	<0.001	0.91 ± 0.05

*Note*: Basic‐adjusted models included age, sex, education, race, and socioeconomic status. Multi‐adjusted models additionally included smoking status, alcohol consumption, physical activity, and Alzheimer's disease‐related polygenic risk score. Least squares (LS) means reflecting mean brain age gap (in years) for each group were estimated from the multi‐adjusted models.

**FIGURE 1:**
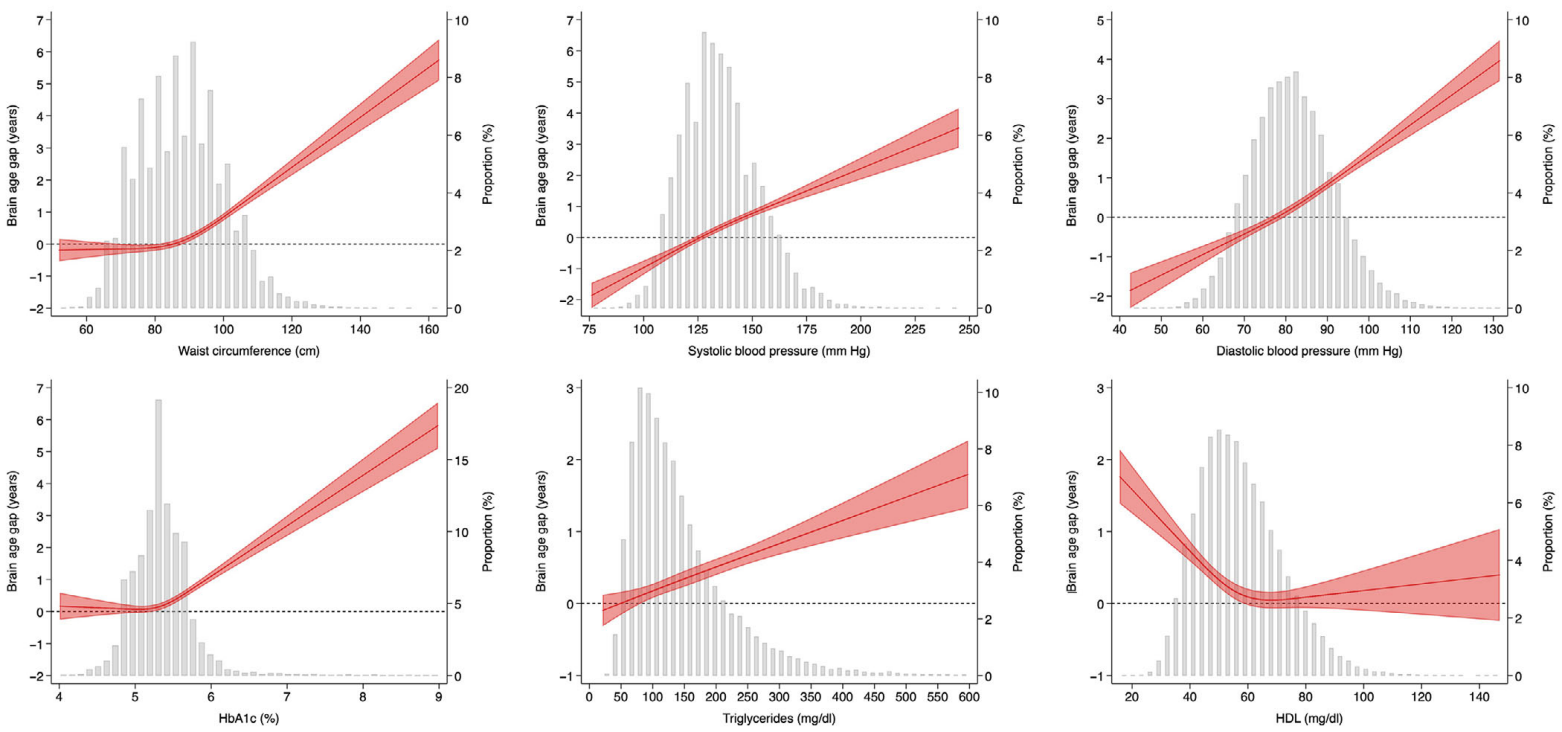
The relationship between continuous components of the MetS definition (waist circumference, systolic and diastolic blood pressure, HbA1c, triglycerides, and HDL cholesterol) and BAG is modeled using restricted cubic splines. The red line and red shaded area represent the least‐squares means and 95% confidence intervals of brain age gap as a function of each MetS‐related measure. Gray bars represent the distribution of each MetS‐related measure in the study population. We observed a significant departure from linearity for waist circumference (*p* <0.001), HbA1c (*p* <0.001), and HDL (*P* <0.001) but not systolic blood pressure (*p* = 0.067), diastolic blood pressure (*p* = 0.056), or triglycerides (*p* = 0.632).

All five components of MetS were also individually related to significantly higher BAG, with the strongest associations observed for hyperglycemia (*β* = 1.08 [0.90, 1.25]) and elevated blood pressure (*β* = 1.10 [0.97, 1.24]). In restricted cubic splines modeling MetS‐related measures as continuous variables (Figure 1), triglycerides, systolic blood pressure, and diastolic blood pressure showed a positive linear association with BAG, whereas non‐linear relationships were observed for waist circumference, HbA1c, and HDL.

**FIGURE 2:**
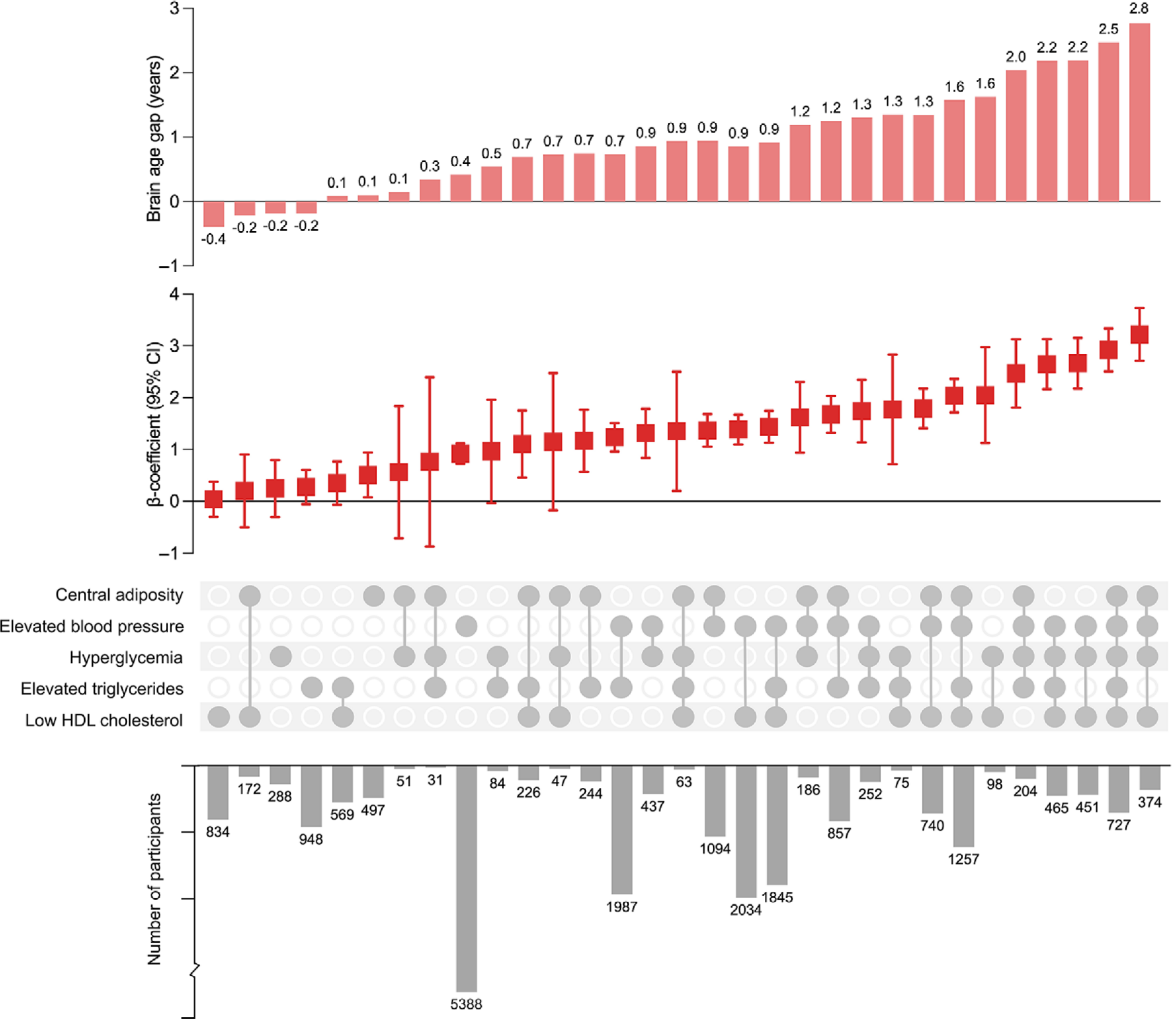
Association between MetS constellations and brain age gap (BAG). Upset plot summarizing the relationship between all possible constellations of the five MetS components and brain age gap. Filled gray dots indicate the presence of each MetS component (central adiposity, elevated blood pressure, hyperglycemia, elevated triglycerides, and low HDL cholesterol), with vertical lines connecting components that co‐occur within a constellation. For each MetS constellation, panels display the estimated BAG (in years), the β‐coefficient and 95% confidence interval for the association with brain age gap, and the number of participants with this profile. Participants with no MetS components (*n =* 4850) were treated as the reference group. See Table S7 for additional details.

We also explored all possible combinations of the five MetS components, finding that the constellations most strongly associated with BAG tended to include hyperglycemia, elevated blood pressure, and low HDL cholesterol (Figure 2, Table S7).

### 3.3 Metabolic signatures associated with MetS and BAG

**FIGURE 3:**
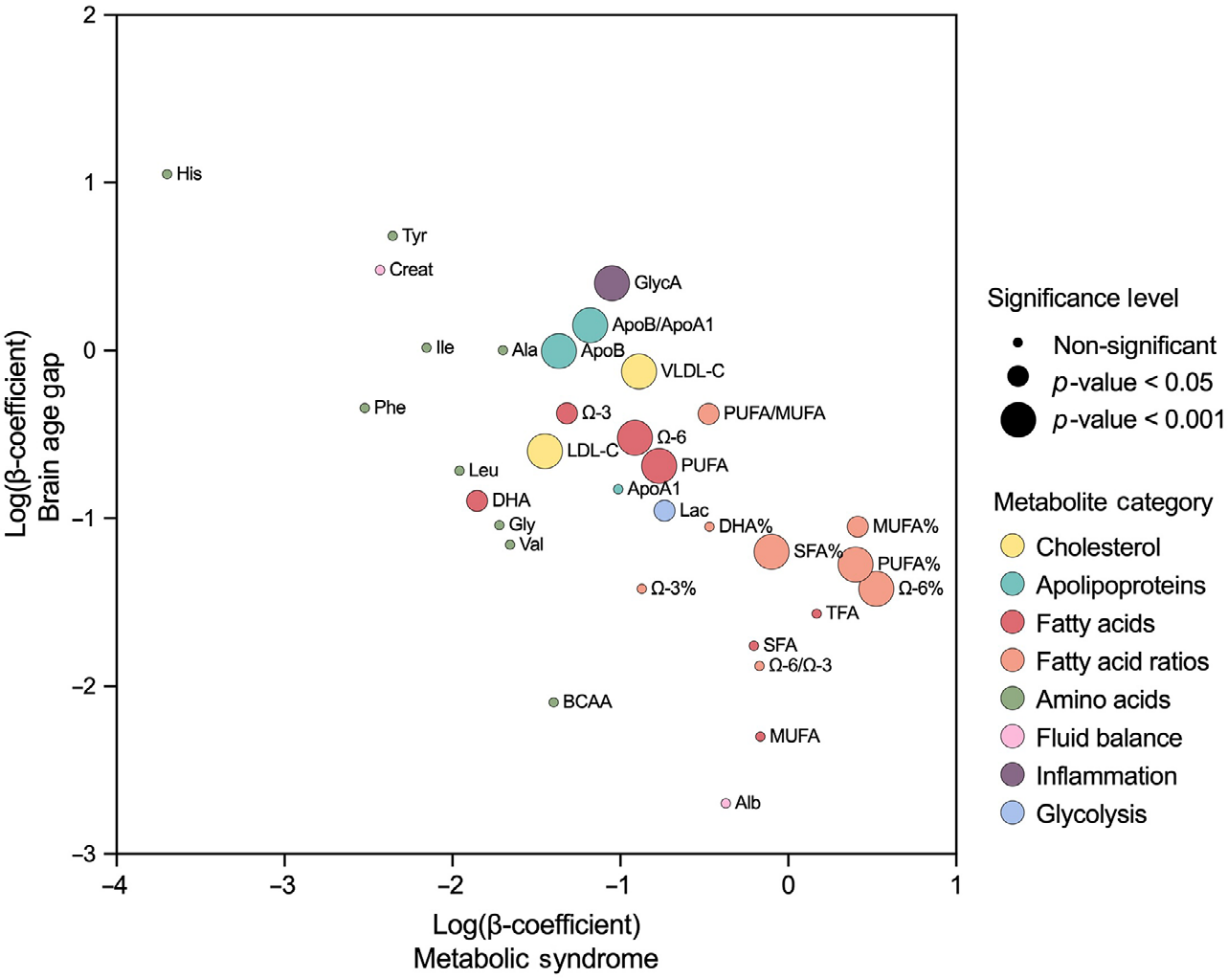
Summary of relationship between clinically validated metabolite biomarkers, metabolic syndrome (MetS), and brain age gap (BAG). Plot shows the strength of each metabolite's association with MetS (*x*‐axis) and BAG (*y*‐axis); 32 of 33 investigated metabolites (all but histidine) were significantly (*p* < 0.0015) associated with MetS. The size of each circle indicates the strength of each metabolite's association with BAG. Models were adjusted for age, sex, education, race, socioeconomic status, smoking status, alcohol consumption, physical activity, and Alzheimer's disease‐related polygenic risk score. See Table S8 for a summary of the β‐coefficients for the associations of each metabolite with MetS and BAG. VLDL‐C, very low‐density lipoprotein cholesterol; LDL‐C, low‐density lipoprotein cholesterol; Apo, apolipoprotein; TFA, total fatty acid; Ω‐3, omega‐3 fatty acid; Ω‐6, omega‐6 fatty acid; PUFA, polyunsaturated fatty acid; MUFA, monounsaturated fatty acid; SFA, saturated fatty acid; DHA, docosahexaenoic acid; Ala, alanine; Gly, glycine; His, histidine; BCAA, branched‐chain amino acid; Ile, isoleucine; Leu, leucine; Val, valine; Phe, phenylalanine; Tyr, tyrosine; Lac, lactate; Creat, creatinine; Alb, albumin; GlycA, glycoprotein acetyls.

Figure 3 illustrates the individual associations of all 33 clinically validated metabolites with MetS and BAG. β‐coefficients for each association are listed in Table S8. A total of 32 metabolites (all but histidine) were significantly associated with MetS, whereas 10 were significantly associated with BAG: low‐density lipoprotein (LDL‐C) and very low‐density lipoprotein (VLDL‐C) cholesterols; apolipoprotein B (ApoB) and the ratio of ApoB to ApoA1 (ApoB/ApoA1); the ratios of omega‐6 fatty acids (Ω‐6%), polyunsaturated fatty acids (PUFA%), and saturated fatty acids (SFA%) to total fatty acids; concentrations of Ω‐6 and PUFAs; and inflammatory glycoprotein acetyls (GlycA).

### 3.4 Identification of metabolite mediators of MetS–BAG association

**FIGURE 4:**
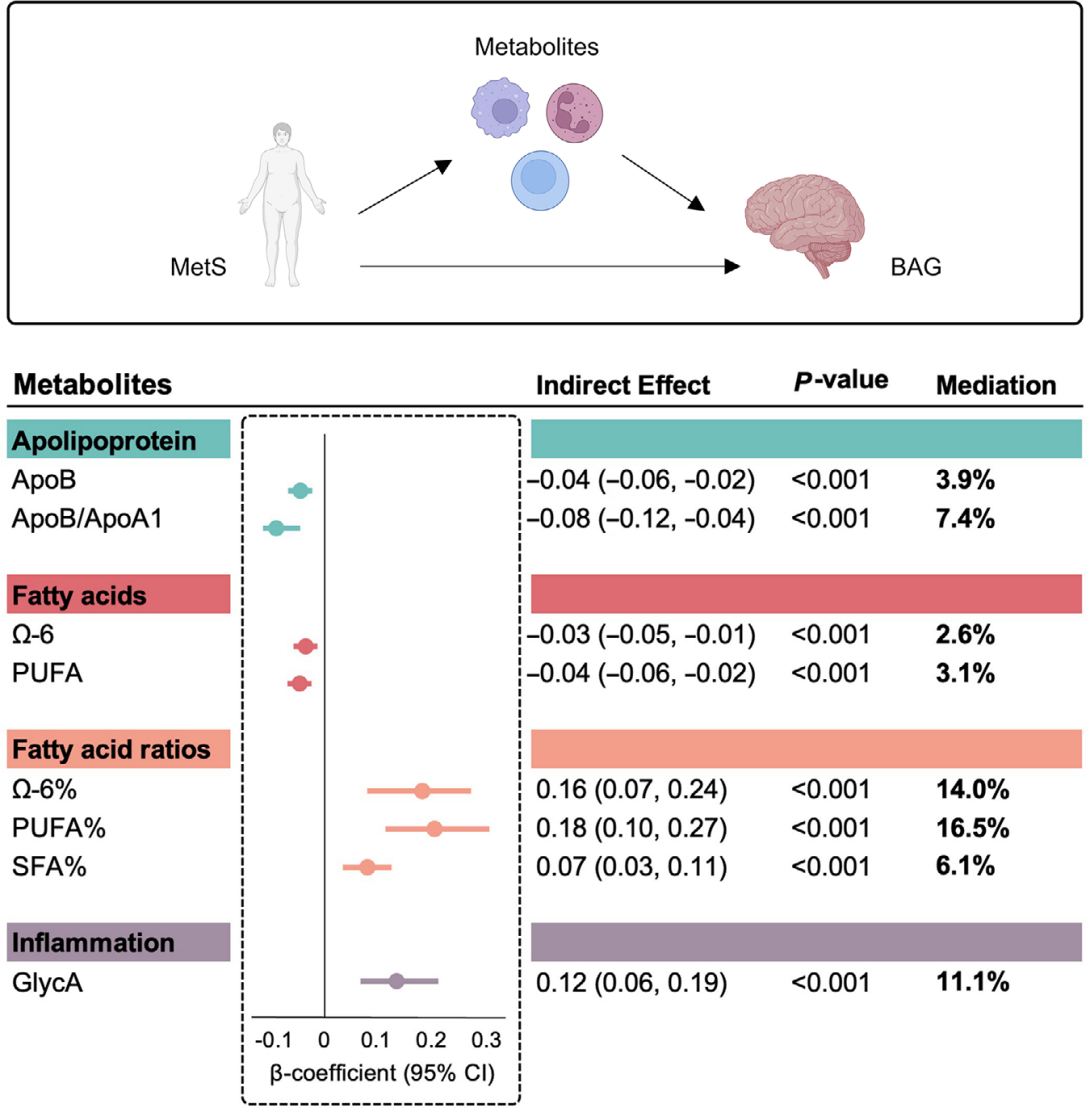
Mediation effect of metabolite biomarkers on the association between metabolic syndrome (MetS) and brain age gap (BAG). The boxed diagram illustrates the hypothesized mediation model for the relationship between MetS, metabolites, and BAG. Beneath, the forest plot and table show the mediation effects and mediation proportions for eight metabolites identified as significant (*p* <0.0015) mediators of the MetS–BAG association. Models were adjusted for age, sex, education, race, socioeconomic status, smoking status, alcohol consumption, physical activity, and Alzheimer's disease‐related polygenic risk score. Apo, apolipoprotein; Ω‐6, omega‐6 fatty acid; PUFA, polyunsaturated fatty acid; SFA, saturated fatty acid; GlycA, glycoprotein acetyl.

In subsequent mediation analysis, eight of the 10 metabolites that were significantly associated with both MetS and BAG emerged as significant mediators of the MetS–BAG association: ApoB, ApoB/ApoA1, Ω‐6, PUFA, Ω‐6%, PUFA%, SFA%, and GlycA (Figure 4, Table S9). The largest proportions of the MetS‐BAG association were mediated by the fatty acid ratios PUFA% (16.5%) and Ω‐6% (14.0%) and the inflammatory marker GlycA (11.1%). The remaining metabolites, namely apolipoproteins and fatty acid concentrations, each mediated between 2.6% and 7.4% of the MetS–BAG association.

**FIGURE 5:**
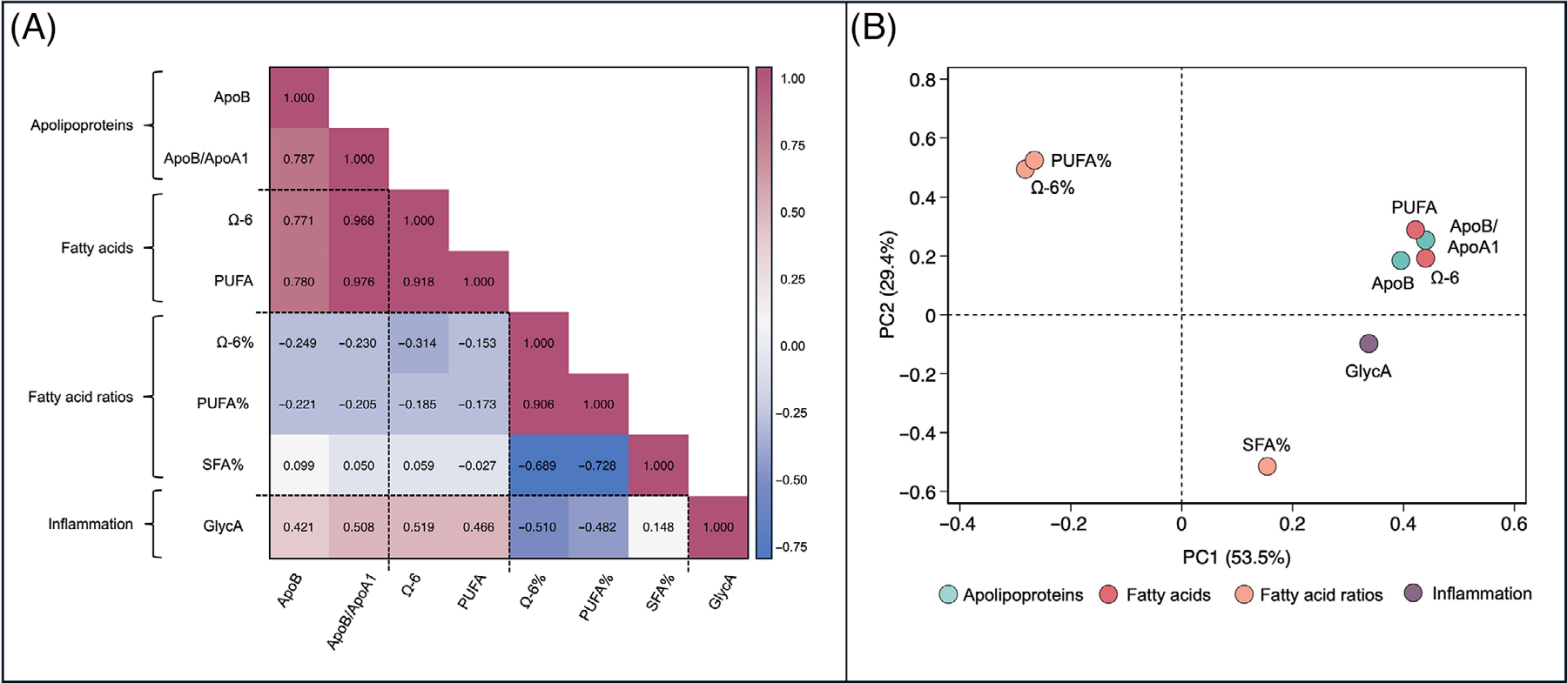
Relationships among the eight metabolites mediating the association between metabolic syndrome (MetS) and brain age gap (BAG). (A) Correlation matrix showing Spearman's correlations between all eight metabolites. (B) Loading plot showing groups of metabolites with similar patterns of variation in principal component analysis. Apo, apolipoprotein; Ω‐6, omega‐6 fatty acid; PUFA, polyunsaturated fatty acid; SFA, saturated fatty acid; GlycA, glycoprotein acetyl.

**FIGURE 6:**
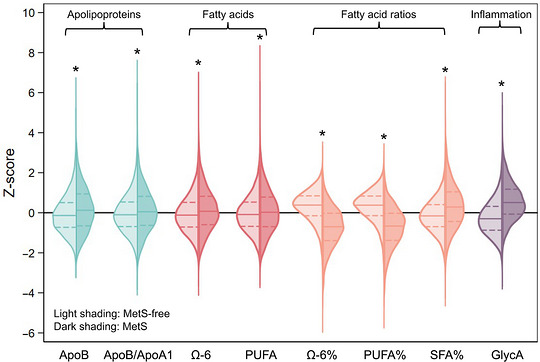
Concentrations of mediating metabolite biomarkers as a function of metabolic syndrome (MetS) status. Apo, apolipoprotein; Ω‐6, omega‐6 fatty acid; PUFA, polyunsaturated fatty acid; SFA, saturated fatty acid; GlycA, glycoprotein acetyl.

All eight metabolites showing significant mediation were also correlated with each other (*p* < 0.0015 for all); strong correlations were observed between members of the same metabolite classes and between apolipoproteins and fatty acids (Figure 5A). In PCA, the metabolites were distributed into four distinct clusters: unsaturated fatty acids and apolipoproteins (ApoB, ApoB/ApoA1, Ω‐6, and PUFA), ratios of unsaturated fatty acids (Ω‐6% and PUFA%), ratios of saturated fatty acids (SFA%), and inflammation (GlycA) (Figure 5B). The concentration of each metabolite differed significantly as a function of MetS status: Ratios of unsaturated fatty acids were higher among MetS‐free participants, and all other metabolites were higher among those with MetS (Figure 6).

### 3.5 Sensitivity analyses

In sensitivity analyses, similar results were obtained when we repeated the analyses using BAG calculated based on brain age estimates from other candidate machine learning models (Table S10) and after using non‐imputed data for covariates (Tables S11 and S12) and accounting for reverse causality by excluding participants with low cognitive performance (Tables S13 and S14), though some metabolites with smaller mediation proportions (ApoB/ApoA1, Ω‐6, and SFA%) no longer met the strict threshold for statistically significant mediation. In sex‐ and age‐stratified analyses, the MetS–BAG association was more pronounced among males and older individuals (Table S15 and S16).

## 4 DISCUSSION

In this large‐scale study integrating neuroimaging and metabolite biomarker data, MetS and all five of its constituent components were related to older brain age in relation to chronological age, and the gap between brain age and chronological age increased with the co‐occurrence of more MetS components. These associations were partially mediated by apolipoproteins, fatty acids, and inflammatory markers.

Using a multimodal MRI‐based measure of brain age, we found that MetS was related to a BAG of 1.26 years. The association between MetS and older brain age followed a dose–response pattern, with BAG increasing monotonically from 0.13 years among participants with a single MetS component to 2.30 years among participants with all five. Each individual component of MetS was also related to older brain age, though the nature of these associations differed. BAG showed a linear increase with higher systolic and diastolic blood pressure, as well as with elevated triglycerides. In contrast, waist circumference (≥90 cm), HbA1c (≥5.5%), and HDL cholesterol (<60 mg/dL) exhibited threshold effects, with BAG increasing only beyond these cutoffs. Having a brain age that exceeds chronological age can be interpreted as an early indicator of a departure from optimal brain health, so it is notable from a public health perspective that even subtle defects in metabolic health are associated with meaningful differences in brain age. Although BAG estimates cannot easily be compared across studies with different methodologies for brain age calculation, contextualization within our own modeling framework suggests that the magnitude of MetS's association with BAG falls somewhere in between that of diabetes (2.29 years)^27^ and unhealthy lifestyle factors like dietary inflammation (0.49 to 0.87 years)^40^ and poor sleep (0.99 years).^39^

Our findings are in line with previous studies linking individual MetS components, including diabetes,^20^, ^21^, ^22^, ^23^, ^24^, ^25^, ^26^, ^27^ elevated blood pressure,^23^, ^24^, ^28^ and obesity,^23^, ^24^, ^29^ to older brain age, as well as another UK Biobank study that used MetS‐related brain morphology to predict accelerated brain aging in participants with stroke and neurodegenerative diseases.^34^ Whereas this previous literature predominantly utilized data on regional brain volumes from T1‐weighted imaging to estimate brain age, our analysis integrated data from six MRI modalities: T2‐FLAIR, T2*, diffusion MRI, resting‐state fMRI, and task‐based fMRI, in addition to T1‐weighted imaging. This multimodal approach is supported by recent findings from another UK Biobank neuroimaging study, which demonstrated that while T1‐weighted imaging is the individual MRI modality with the highest predictive accuracy for brain age estimation, the best model performance is achieved when multiple MRI modalities are combined.^24^ Moreover, through the inclusion of other MRI modalities in addition to T1‐weighted imaging, our brain age estimate reflects a wide range of brain phenotypes, including not only regional brain volumes but also markers of vascular brain health (from T2‐FLAIR, T2*, and diffusion MRI) and functional connectivity (from resting‐state and task‐based fMRI). The incorporation of several IDP markers of vascular brain health – including white matter hyperintensities, microbleeds, and measures of white matter microstructural integrity like mean diffusivity, and fractional anisotropy – into our brain age estimate is especially relevant to capture the full extent of MetS and its constituent vascular risk factors on brain health.

Our study extends the existing literature on MetS and brain‐related outcomes through the incorporation of plasma metabolite biomarkers to investigate the biological mechanisms by which MetS might impact the brain. One cluster of metabolites that emerged as significant mediators of the MetS–BAG association was apolipoproteins (ApoB and ApoB/ApoA1), which accounted for 3.9% to 7.4% of the association. ApoB and the ApoB/ApoA1 ratio are markers of atherogenicity,^46^, ^47^ pointing to atherosclerosis as a potential mechanism connecting MetS and brain aging. The MetS–BAG association was also mediated 2.6% to 3.1% by fatty acid concentrations (Ω‐6, PUFA) and 6.1% to 16.5% by fatty acid ratios (Ω‐6%, PUFA%, SFA%). Fatty acids – especially PUFAs – are highly abundant in the brain and play a major role in maintaining the structure and function of neurons and glia,^48^ and MetS‐related alterations in fatty acid metabolism may disrupt these processes, contributing to poorer brain health. Finally, the inflammatory marker GlycA mediated 11.1% of the MetS‐BAG association, consistent with a large literature linking MetS and its components to inflammation and, in turn, linking systemic inflammation to neuropathologies ranging from cerebrovascular disease to brain amyloid accumulation to neurodegeneration.^49^, ^50^, ^51^ However, with mediation proportions ranging from 2.6% to 16.5%, it is important to note that a large part of the MetS–BAG association remains unexplained by the metabolites examined here.

The identified metabolites may interact with one another in complex ways. In PCA and heatmapping analysis, metabolites reflecting the concentrations of apolipoproteins and unsaturated fatty acids (PUFA, Ω‐6, ApoB, ApoB/ApoA1) clustered together and showed strong correlations, suggesting that these may operate through overlapping or synergistic pathways – though this interpretation is speculative given the cross‐sectional and observational nature of the data. Other metabolites like the inflammatory marker GlycA clustered separately, suggesting potentially distinct pathways. It is also possible that the identified metabolites are reflective of broader metabolic dysregulation rather than specific mechanistic pathways. More detailed examination of the mechanisms connecting metabolic and brain health is a rich area for future investigation.

### 4.1 Strengths and limitations

The strengths of this study include the large sample size, the availability of objective measures to ascertain MetS, the use of multimodal brain MRI data to estimate brain age, and the inclusion of a panel of clinically validated plasma metabolite biomarkers to explore potential biological mechanisms connecting MetS to accelerated brain aging. That said, some limitations should be acknowledged. First, the UK Biobank suffers from well‐documented healthy volunteer bias, with a study population that is substantially healthier, more socioeconomically advantaged, and less diverse than the general UK population.^52^, ^53^ An additional layer of selection bias was unavoidably introduced in the restriction of our analytical samples to participants with available neuroimaging and metabolite biomarker data – a group that is younger, more highly educated, and metabolically healthier compared to the overall UK Biobank sample (Table S17). These overlapping sources of selection likely compress variance in both the MetS exposure and BAG outcome, contributing to a potential underestimation of the association between MetS, its components, and brain age. The relative ethnic homogeneity of our sample also limits generalizability to non‐European populations, among whom MetS prevalence, metabolite profiles, and patterns of brain aging may differ. Another limitation is potential reverse causality, insofar as accelerated brain aging may contribute to the development of MetS by making it more difficult to manage medical conditions and adhere to a healthy lifestyle. We accounted for this in sensitivity analyses excluding participants with possible cognitive impairment, and associations remained consistent with the original analyses, suggesting that reverse causality did not substantially impact our results. Similarly, the concurrent measurement of MetS and metabolite biomarkers at baseline is a limitation, as it precludes formal verification of causal directionality in the mediation model. Finally, brain MRI data were available at only one time point, limiting our ability to investigate the longitudinal relationship between MetS and brain aging. Ongoing collection of repeat brain MRI scans in the UK Biobank will allow future studies to determine whether MetS is associated with an accelerated pace of brain aging over time.^54^

## 5 CONCLUSION

In conclusion, this study highlights the association between MetS and accelerated brain aging and provides evidence that this association may be driven in part by elevated levels of systemic inflammation and alterations in apolipoprotein and fatty acid metabolism. Taken together, our findings shed light on the biological mechanisms linking MetS and the brain and highlight MetS and its components as potential targets for interventions to promote healthy brain aging.

## AUTHOR CONTRIBUTIONS

Abigail Dove, Jiao Wang, and Weili Xu contributed to the conception and design of the study. Abigail Dove conducted the statistical analyses, performed the literature search, and drafted the first version of the manuscript. Rongrong Yang, Sakura Sakakibara, Zoe Arvanitakis, Andrea L. C. Schneider, and Rebecca F. Gottesman interpreted the data and provided critical revisions to the manuscript. All authors made a significant contribution to finalizing the manuscript and approved the final version for publication.

## CONFLICT OF INTEREST STATEMENT

Abigail Dove, Jiao Wang, Rongrong Yang, Sakura Sakakibara, Andrea L. C. Schneider, Rebecca F. Gottesman, and Weili Xu have nothing to disclose. Zoe Arvanitakis reports receiving consulting fees from Novo Nordisk, Amylyx, Eisai, California Institute of Regenerative Medicine, and Summus.Author disclosures are available in the Supporting Information.

## CONSENT STATEMENT

All participants in the UK Biobank provided informed consent at baseline and prior to the MRI scan, and the data collection procedures were approved by the National Health Services (NHS) National Research Ethics Service (Ref 11/NW/0382). Use of the data for the present analyses was additionally approved by the Regional Ethical Review Board in Stockholm (2024‐00520‐01). All data used in this study were obtained from the UK Biobank through application 67048 (PI: Weili Xu).

## Supporting information




**Supplementary Information**: alz71563‐sup‐0001‐SupMat.docx

**Supplementary Information**: alz71563‐sup‐0002‐ICMJE.pdf
